# Beef Cattle Body Weight Estimation Based on Dual-View RGB Images

**DOI:** 10.3390/ani16162532

**Published:** 2026-08-13

**Authors:** Ziruo Li, Yadan Zhang, Chong Yao, Ying Han, Zenglong Song, Xueting Zeng, Xiaocong Li, Gang Liu

**Affiliations:** 1 Key Laboratory of Smart Agriculture Systems, Ministry of Education, China Agricultural University, Beijing 100083, China; liziruo123@cau.edu.cn (Z.L.); lyzhzyd@163.com (Y.Z.); yaochong@cau.edu.cn (C.Y.); hanyingxx@163.com (Y.H.); 19931055739@163.com (Z.S.); xueting_cau@163.com (X.Z.); chatlxc@163.com (X.L.); 2 Key Laboratory of Agricultural Information Acquisition Technology, Ministry of Agriculture and Rural Affairs of China, China Agricultural University, Beijing 100083, China

**Keywords:** body weight estimation, dual-view RGB images, instance segmentation, dual-view feature fusion, non-contact measurement

## Abstract

Non-contact body weight (BW) estimation provides a low-stress and low-cost approach for precision beef cattle management, but single-view RGB images may not fully capture body-shape information. This study proposed a practical dual-view RGB framework for cattle BW estimation. A total of 3210 paired top-view and side-view RGB images were collected from 107 Simmental beef cattle with BW ranging from 169 to 980 kg. An EMA-enhanced YOLO11n-seg model was adopted to improve cattle foreground extraction, and a two-stream CBAM-ResNet50-SE network was constructed to learn dorsal and lateral morphological features for BW regression. The EMA-YOLO11n-seg model achieved mAP@0.5 values of 99.18% and 98.35% for top-view and side-view images, respectively. On the test set, the proposed BW estimation model achieved an MAE of 14.96 kg, an RMSE of 17.86 kg, and an R^2^ of 0.85. The model also showed stable performance across different growth stages and posture conditions. Adaptation experiments using a Sanhe cattle dataset further demonstrated the adaptability of the proposed framework. These results suggest that the practical dual-view RGB framework developed in this study provides an effective solution for non-contact beef cattle BW estimation under fixed image-acquisition conditions.

## Simple Summary

Accurate body weight information is important for beef cattle growth monitoring, feeding management, health assessment, and marketing decisions. Traditional weighing methods often require cattle to be driven onto scales, which is labor-intensive and may cause stress to the animals. This study developed a non-contact method for estimating beef cattle body weight using ordinary color images captured from the top and side of each animal. In total, 3210 paired images were collected from 107 Simmental beef cattle. A model was used to separate the cattle body from the background and then combine body-shape information from both views to estimate body weight. The proposed method achieved an average error of 14.96 kg on the test set and showed stable performance for cattle at different growth stages and in different postures. The results suggest that dual-view image analysis can help farmers monitor beef cattle body weight more conveniently, reduce animal handling stress, and support more efficient precision livestock management.

## 1. Introduction

In beef cattle production, body weight is an important indicator for growth monitoring [1], feeding management [2], health assessment [3], and production efficiency analysis [4]. It also provides a basis for feed optimization [5] and slaughter decision-making [6]. Therefore, timely and accurate acquisition of beef cattle body weight information is of great importance [7,8]. Traditional beef cattle weighing mainly relies on static scales or weighing platforms. Although these methods can provide high measurement accuracy, they require manual driving and restraint of cattle, are labor-intensive and time-consuming, and may induce stress responses, thereby affecting animal welfare and production performance [2,9]. Another approach is to manually measure body size parameters and establish regression models for weight estimation. However, this method still requires close contact with animals, involves high labor intensity, and is easily affected by operator experience, animal posture, and measurement environment [10,11].

With the development of computer vision and deep learning technologies, new solutions have been provided for non-contact beef cattle weight estimation [12]. Existing studies usually first extract body size parameters related to body weight from images or three-dimensional data, such as body height [13], body width [14], and oblique body length [15], and then establish body weight regression models based on these parameters. Common regression methods include linear regression [15], support vector machine [16], and neural networks [17]. However, these methods often require two-stage estimation, which can lead to accumulated errors and reduce weight estimation accuracy.

Subsequent studies have used RGB-D images or three-dimensional point clouds for beef cattle weight estimation to obtain richer spatial structure and volume information, thereby improving estimation accuracy. Song et al. [18] collected top-view RGB-D images of 20 Simmental beef cattle weighing 300–500 kg for body weight estimation. Yao et al. [19] proposed a Dyrep-optimized two-stream asymmetric feature extraction network based on RGB-D images, which separately modeled the feature differences between RGB and depth images for beef cattle live weight estimation and achieved an MAE of 16.20 kg on the validation set. Hou et al. [14] used side-view point cloud data of beef cattle and adopted a PointNet++ model for body weight regression, achieving a MAPE of 3.2% and an RMSE of 10.2 kg. These techniques usually require the target animal region to be extracted from the original images before morphological feature extraction and body weight regression are performed on the segmented images. Existing cattle body region extraction methods include manually generated segmentation masks or manual-assisted contour extraction [20,21], traditional image segmentation methods based on thresholding or template matching [22,23], and deep learning-based semantic segmentation and instance segmentation networks [17,24]. Among them, U-Net [19], Mask R-CNN [25], and YOLO-series models [26] have been widely used for livestock target region extraction and contour segmentation. Target region segmentation has also been proven to improve the accuracy of body weight estimation [19,21,22]. Although RGB-D images and point clouds can provide three-dimensional information, they also have limitations, such as high equipment cost, complex processing procedures, and susceptibility to noise and environmental interference. Therefore, this study explores beef cattle weight estimation based on RGB images, aiming to reduce hardware cost and data processing complexity while improving estimation accuracy and practical applicability by using complementary morphological information from two views.

This paper aims to improve the accuracy and stability of non-contact beef cattle BW estimation under fixed weighing-channel conditions by combining instance segmentation and dual-view feature fusion. The specific work includes: (1) constructing an EMA-YOLO11n-seg instance segmentation model to automatically extract beef cattle foreground regions from top-view and side-view RGB images, and verifying the contribution of instance segmentation to improving weight estimation accuracy; (2) constructing a two-stream CBAM-ResNet50-SE weight estimation network to fuse complementary morphological features from the dorsal and side views of beef cattle, with a focus on evaluating its performance in terms of accuracy (MAE, RMSE, and R^2^); and (3) evaluating the stability of the proposed method using cattle at different growth stages and under different posture conditions, and further assessing its adaptability on a Sanhe cattle dataset.

## 2. Materials and Methods

### 2.1. Beef Cattle Data Acquisition

**Figure 1:**
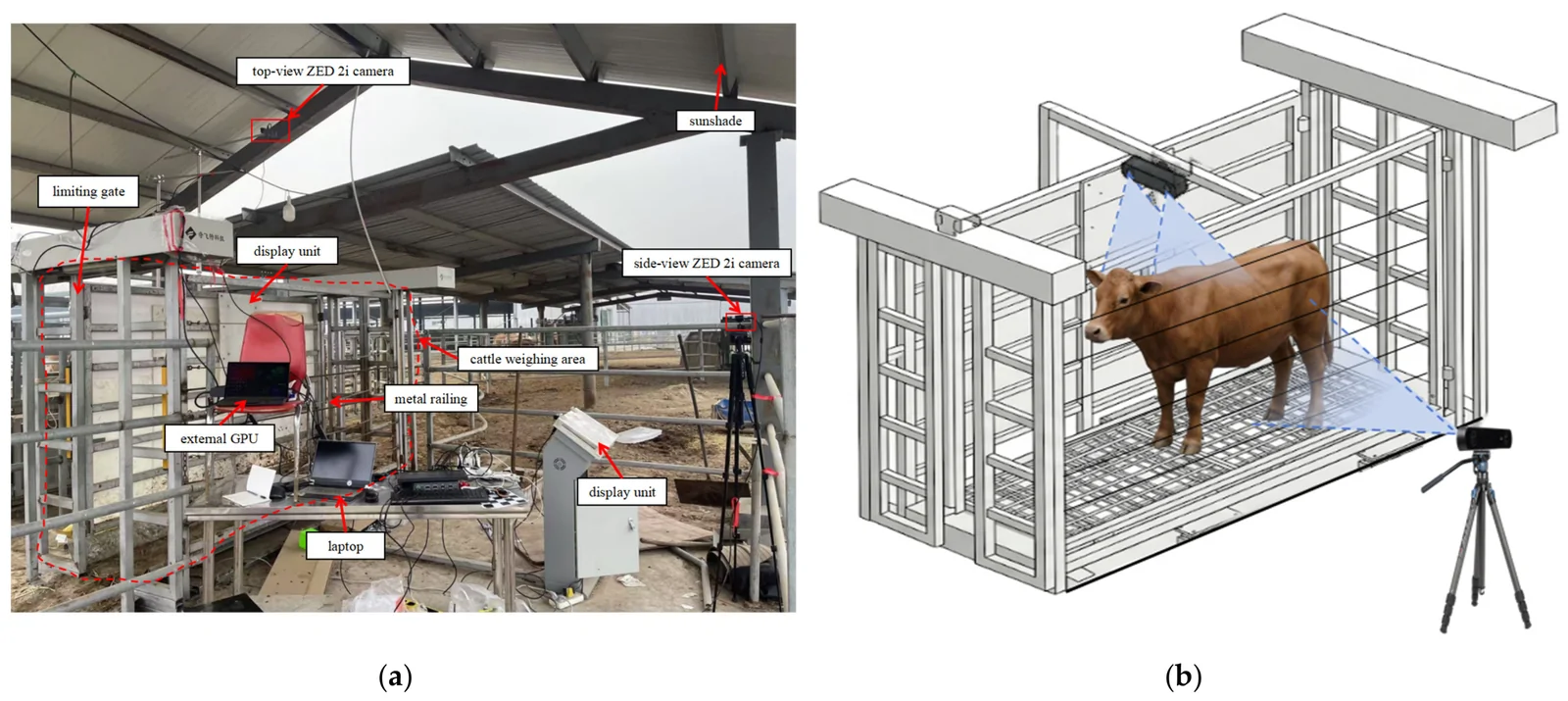
Dual-view RGB image-acquisition system for beef cattle body weight estimation: (**a**) schematic diagram of the acquisition setup; (**b**) field setup of the image-acquisition system.

On 3–6 January 2025, dual-view RGB video data of Simmental beef cattle were collected at Dingyuan Breeding Cattle Co., Ltd., located in Zhoukou City, Henan Province, China (longitude 114.70, latitude 33.64). In the experiment, weight measurements and dual-view RGB images were collected from 107 Simmental beef cattle, aged from 3 to 20 months, using two ZED 2i cameras (Stereolabs, Paris, France), while body weight was recorded with a static platform scale (XK3190-A17E, YAOHUA, Shanghai, China). The dual-view RGB image-acquisition system and field setup are shown in Figure 1. The side-view camera was installed at a height of 1.5 m above the ground and approximately 2.8 m from the weighing channel, while the top-view camera was installed at a height of 2.97 m above the ground. The top-view camera was vertically oriented downward, and the side-view camera was installed perpendicular to the weighing channel. Both cameras were fixed during data acquisition, with a frame rate of 15 fps and an image resolution of 2208 × 1242 pixels. The raw videos were saved in SVO format and parsed using the Stereolabs ZED SDK. The top-view and side-view frames were synchronized according to acquisition timestamps, and 30 paired frames were extracted for each animal when the cattle body was clearly visible in both views. Blurred frames, incomplete body images, and severely occluded frames were excluded. Most cattle in the Simmental dataset had clear coat patterns and smooth body surfaces, which facilitated visual feature extraction under the fixed acquisition setup. This uniformity helps minimize external variability, allowing the model to focus more effectively on extracting and predicting body-shape features. Data collection was carried out in a shaded weighing channel using fixed dual-view camera systems to reduce the influence of ambient light variation. The dataset covered a body weight range from 169 to 980 kg. To avoid data leakage and ensure a fair evaluation of model generalization, the dataset was divided into training, validation and test sets according to cattle identity rather than image-level random splitting. Finally, 68 cattle with 2040 image pairs were used for training, 17 cattle with 510 image pairs were used for validation, and 22 cattle with 660 image pairs were used for testing. The annotated images for instance segmentation followed the same cattle-level division strategy to avoid data leakage. In addition, a Sanhe cattle dataset collected in October 2023 in Hulunbuir, Inner Mongolia, China (longitude 120.00, latitude 49.34), using two Microsoft Azure Kinect DK cameras (Washington, DC, USA), with body weight recorded during data acquisition, was used to further evaluate the adaptability of the proposed framework to another beef cattle breed. This dataset contained 68 Sanhe cattle with 680 dual-view RGB image pairs, covering a body weight range of 274–515.5 kg. Table 1 summarizes the cattle characteristics and data distribution of the constructed dataset. The cattle were categorized into four growth stages, including the calf stage, early fattening stage, middle fattening stage, and late fattening stage. Detailed information on the number of cattle, body weight range, mean body weight, and the distribution of training and test samples is also provided. Detailed information on the feeding management of the experimental cattle and the growth-stage classification criteria is provided in Appendix A.1.

### 2.2. Camera Calibration and Scale Normalization

In order to reduce imaging-scale differences caused by camera installation and shooting distance, the top-view and side-view cameras were calibrated separately using the same checkerboard with 60 mm × 60 mm squares. For the Simmental cattle dataset and the Sanhe cattle dataset, the ZED 2i and Microsoft Azure Kinect DK cameras were independently calibrated, respectively. Checkerboard images with different positions and poses were used to estimate camera intrinsic parameters and distortion coefficients, and the RGB images were corrected for distortion. Based on checkerboard corners near the main cattle imaging region, reference-plane scale normalization was further performed to map the top-view and side-view images to a consistent physical scale. The normalized images were then resized and padded to 640 × 640 pixels for segmentation and body weight estimation. The calibration model and scale normalization process are provided in Appendix A.2.

### 2.3. Image Pre-Processing

**Figure 2:**
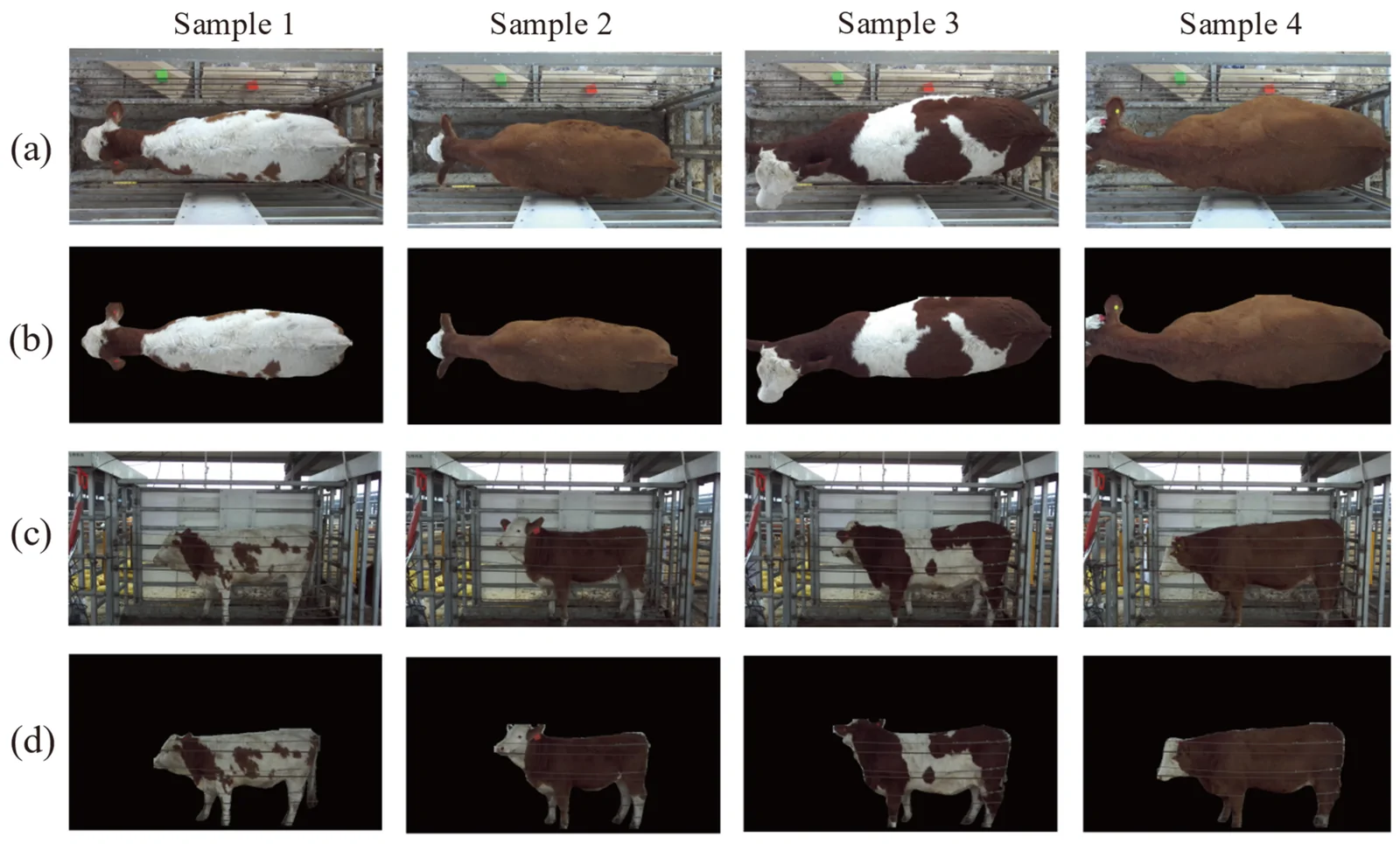
Representative dual-view RGB images of beef cattle before and after instance segmentation. Sample 1–4 denote four representative beef cattle samples. (**a**) Original top-view images; (**b**) segmented top-view images; (**c**) original side-view images; (**d**) segmented side-view images.

The original dual-view RGB images consisted of top-view and side-view images with a resolution of 2208 × 1242 × 3. Since the images were collected in a real farm, they contained complex background information, such as railings, ground regions, and surrounding equipment, which could interfere with body weight estimation. To obtain accurate cattle foreground regions, 1500 pairs of dual-view RGB images were manually annotated using Labelme (version 5.7.0) to generate instance segmentation labels. The number of annotated images varied among individual cattle due to differences in captured poses and image quality. The annotated images were divided according to cattle identity to avoid information leakage between different subsets. After training and comparing different segmentation models, the best-performing EMA-YOLO11n-seg model was selected to segment all images. The generated cattle masks were used to remove background regions, and representative examples of the original and segmented dual-view RGB images are shown in Figure 2. Finally, the processed top-view and side-view images were paired according to the same individual and sampling moment and used as inputs to the dual-stream body weight estimation network. To evaluate the effect of segmentation, comparative experiments were conducted using both original and segmented images, as described in Section 3.2.

**Figure 3:**
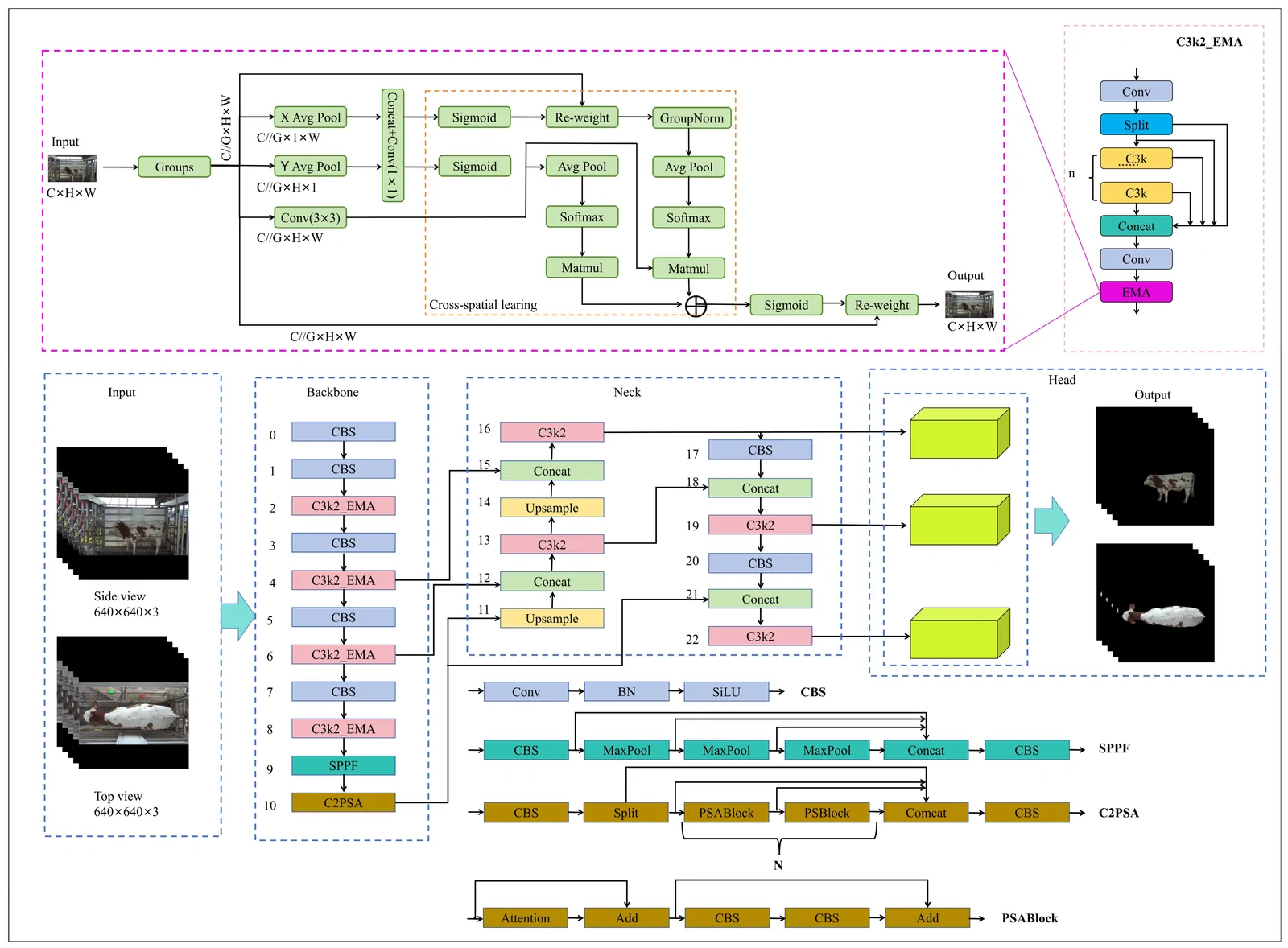
Architecture of the proposed EMA-YOLO11n-seg model for beef cattle instance segmentation.

In this paper, the backbone of YOLO11n-seg was improved by replacing the original C3k2 module with a C3k2-EMA module integrated with the Efficient Multi-Scale Attention (EMA) [27] mechanism, so as to improve the extraction of cattle body-edge and local contour features. Body weight estimation depends strongly on body contour information, especially the abdominal curve, dorsal line, and trunk boundary in side-view images. Therefore, improving boundary segmentation can help reduce the effects of background interference and contour loss on subsequent weight regression. The EMA module enhances the interaction between channel and spatial features through multi-scale feature modeling, with only a slight increase in model complexity. After being embedded into the C3k2 module, the network can better focus on weight-related regions, such as the back, abdomen, and lateral body edges, thereby improving foreground segmentation completeness and boundary continuity. The overall structure of EMA-YOLO11n-seg is shown in Figure 3.

### 2.4. Dual-View Beef Cattle Weight Estimation Model Based on Two-Stream CBAM-ResNet50-SE Network

In this study, a two-stream CBAM-ResNet50-SE network was used for beef cattle body weight estimation. The model takes the segmented top-view and side-view images as paired inputs, extracts body-shape features from different views, and performs body weight regression through feature fusion. The top-view branch mainly extracts dorsal morphological features, such as back width, back contour, and trunk outline, while the side-view branch mainly extracts lateral body-shape features, such as body length, body height, dorsal line, abdominal curve, and trunk boundary. Since a single view cannot fully represent cattle body shape, dual-view inputs can complement morphological features from different perspectives and provide a more complete representation for body weight estimation.

It should be noted that both CBAM [28] and SE are existing attention modules. They were introduced not to propose a new attention mechanism or network structure, but to adapt the feature extraction and feature fusion processes to the requirements of dual-view cattle body weight estimation. Body weight estimation mainly relies on morphological features such as body width, body length, back contour, abdominal curve, and trunk boundary, whereas head posture, limb regions, and local texture variations may introduce weakly related interference. Therefore, CBAM was embedded into the top-view and side-view ResNet50 branches to enhance body-shape-related feature responses from both channel and spatial dimensions. Then, an SE module was introduced after dual-view feature concatenation to adaptively recalibrate the fused channel features and adjust the relative contributions of top-view and side-view features in body weight regression. Thus, CBAM is mainly used for single-view morphological feature enhancement, while SE is mainly used for dual-view fused feature recalibration, corresponding to the feature extraction and feature fusion stages, respectively.

The segmented top-view and side-view images were resized to 640 × 640 × 3 and then input into the two ResNet50 feature extraction branches. Each branch extracted high-level morphological features through the Stem module and four residual stages, generating a feature map F3 with a size of 40 × 40 × 2048. To enhance body-shape-related feature responses, CBAM was introduced into each ResNet50 branch. CBAM consists of a channel attention module and a spatial attention module. The channel attention module uses global average pooling and global max pooling to generate channel descriptors, which are processed by a shared MLP and a Sigmoid function to obtain the channel attention weight Mc, expressed as follows:

(1) Mc(F)=σ(MLP(AvgPool(F))+MLP(MaxPool(F)))

where σ is the activation function.

The SA module further highlights spatial regions related to cattle body shape. It performs average pooling and maximum pooling operations along the channel dimension on the enhanced top-view feature map of beef cattle, and concatenates them to obtain effective features highlighting the location area. Then, through a 7 × 7 convolution and processing with the Sigmoid activation function, the spatial attention Ms is obtained, and its expression is:

(2) $$Ms(F^{\prime })=\sigma (f^{7\times 7}([AvgPool(F^{\prime }),MaxPool(F^{\prime })]))$$

where $f^{7\times 7}$ represents 7 × 7 convolution operation.

The CBAM attention mechanism is first applied to the channel dimension of the enhanced top feature map F3 of beef cattle, and then to the spatial dimension. That is, F3 is first point-multiplied by the channel attention feature map Mc to obtain the refined feature map, then $F3^{\prime \prime }$ is element-wise multiplied by the spatial attention feature map Ms to get the final output feature $F3^{\prime \prime }$ of the beef cattle’s top.

The segmented top and side feature maps of beef cattle are separately input into the two-stream CBAM-ResNet50 network to extract features from the back and abdomen of the cattle. The enhanced top features and enhanced side features of the beef cattle are then concatenated to obtain a multi-view beef cattle fusion feature map.

Then, the enhanced features extracted from the top-view and side-view branches were concatenated to obtain dual-view fused features. Considering that the contributions of top-view and side-view features to body weight estimation may vary across growth stages and posture conditions, a lightweight SE module was introduced after feature fusion [29]. Through squeeze, excitation, and scaling operations, the SE module recalibrates the fused features along the channel dimension and adaptively adjusts the importance of different feature channels, thereby enhancing weight regression-related feature representation and reducing the influence of weakly relevant features.

**Figure 4:**
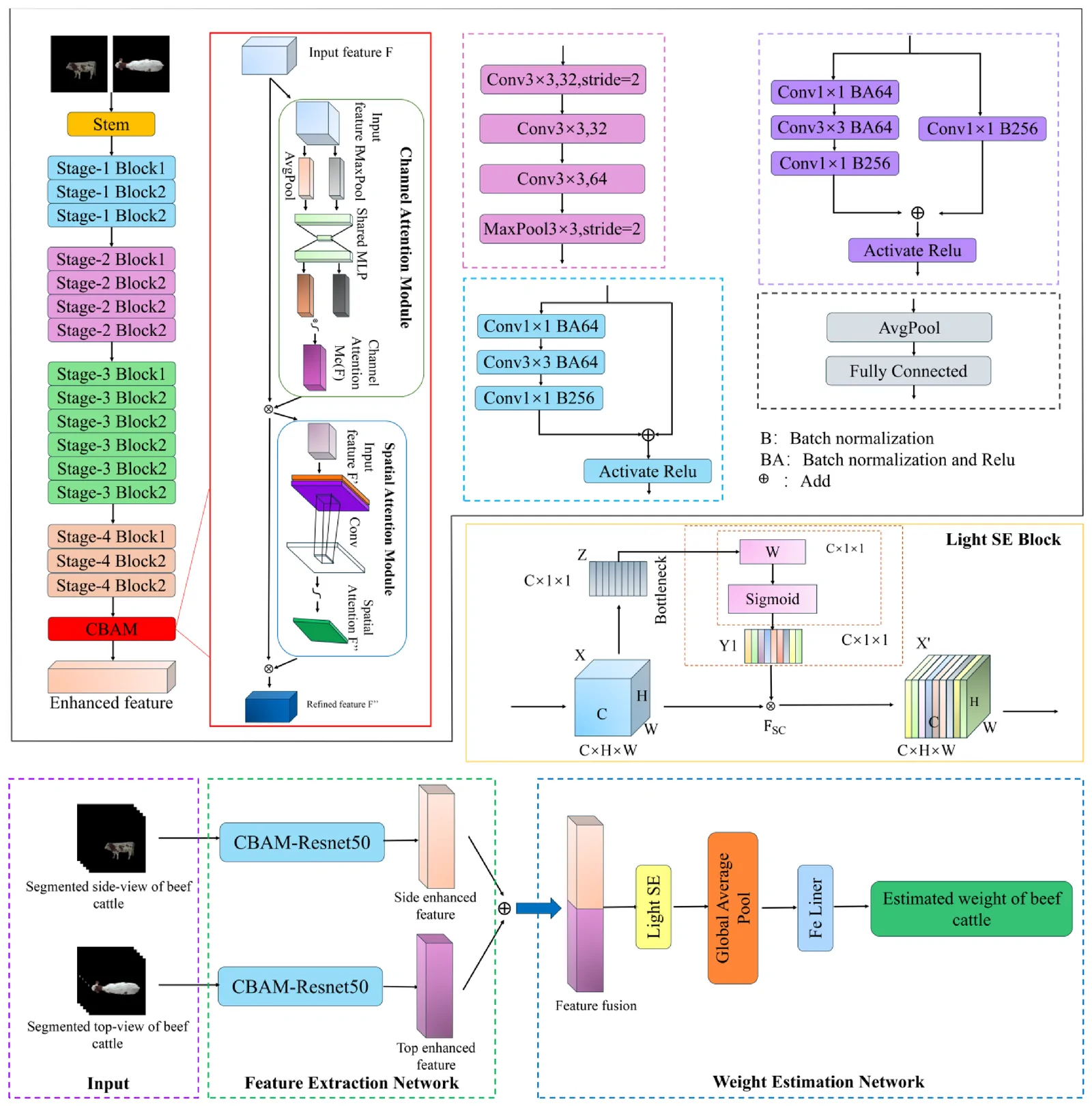
Structure diagram of CBAM-ResNet50-SE beef cattle body weight estimation model.

The enhanced multi-view fused feature map $X^{\prime }$ of beef cattle undergoes the global average pool operation in sequence to compress the features in the spatial dimension and extract more representative global features. Then, a fully connected layer (Fc) is used to process global features, mapping them to the dimension related to beef cattle body weight estimation. Finally, the estimated body weight value of beef cattle is output. The structure diagram of the CBAM-ResNet50-SE beef cattle body weight estimation model is shown in Figure 4.

### 2.5. Experimental Platform and Evaluation Metrics

All experiments were conducted in Python 3.9 with CUDA 11.8. For instance segmentation, a YOLO11-based model was trained using the Ultralytics framework for 100 epochs with a batch size of 64, 8 dataloader workers, SGD optimization, and automatic mixed precision enabled. For body weight regression, the model was implemented in PyTorch 2.3.1 and trained for 100 epochs using AdamW with an initial learning rate of 0.001, a weight decay of 0.01, a batch size of 64, and mixed-precision training enabled.

To comprehensively evaluate the model performance, this study conducted two sets of experiments: instance segmentation and body weight regression. For the instance segmentation task, Precision (P), Recall (R), mAP@0.5, mAP@0.5:0.95, and Dice Mask IoU were used to evaluate the segmentation performance of EMA-YOLO11n-seg on the top-view and side-view beef cattle image datasets. The definitions of these evaluation metrics are as follows:

(3) $$P=\frac{TP}{TP+FP}\times 100\%$$

(4) $$R=\frac{TP}{TP+FN}\times 100\%$$

(5) $$mAP@0.5=\frac{1}{N}\sum_{i=1}^{N}AP_{i,0.5}\times 100\%$$

(6) $$mAP@0.5:0.95=\frac{1}{N}\sum_{i=1}^{N}\frac{1}{10}\sum_{t=0.50}^{0.95}AP_{i}\times 100\%$$

(7) $$Dice=\frac{2\left|P_{m}\cap G_{m}\right|}{\left|P_{m}\right|+\left|G_{m}\right|}\times 100\%$$

(8) $$MaskIoU=\frac{\left|P_{m}\cap G_{m}\right|}{\left|P_{m}\cup G_{m}\right|}\times 100\%$$

*TP* is the number of correctly detected beef cattle instances; *FN* is the number of missed beef cattle instances; *FP* is the number of background or non-target regions incorrectly detected as beef cattle; $AP_{i,0.5}$ denotes the average precision of the *i*-th category at an IoU threshold of 0.5; and $AP_{i,t}$ denotes the average precision of the *i*-th category at an IoU threshold of t. N is the number of object categories. $P_{m}$ and $G_{m}$ denote the predicted mask and ground-truth mask, respectively.

In the body weight regression task, this study uses mean absolute error (MAE) to measure the error between the estimated and true values of beef cattle body weight, and root mean square error (RMSE) to evaluate the stability of the beef cattle body weight prediction method. The coefficient of determination (R-squared, R^2^) is used to assess the ability of the regression model to explain the variation in beef cattle body weight. The relative error rate (RER) is used to measure the deviation between the predicted body weight and true body weight of a single beef cattle sample, and the mean relative error (MRE) is used to calculate the average of relative error rates across multiple beef cattle samples, i.e., the overall prediction deviation of the model for a set of beef cattle samples. Meanwhile, Parameters and FLOPs are used to measure the size and inference efficiency of the beef cattle body weight estimation model. The definitions of evaluation metrics for the beef cattle body weight regression task are as follows:

(9) $$MAE=\frac{1}{n}\sum_{i=1}^{n}\left|y_{i}-{\hat{y}}_{i}\right|$$

(10) $$RMSE=\sqrt{\frac{1}{n}\sum_{i=1}^{n}{\left(y_{i}-{\hat{y}}_{i}\right)}^{2}}$$

(11) $$R^{2}=1-\frac{\sum_{i=1}^{n}{\left(y_{i}-{\hat{y}}_{i}\right)}^{2}}{\sum_{i=1}^{n}{\left(y_{i}-\bar{y}\right)}^{2}}$$

(12) $$RER=\frac{\left|{\hat{y}}_{i}-y_{i}\right|}{y_{i}}\times 100\%$$

(13) $$MRE=\frac{1}{n}\sum_{i=1}^{n}\left|\frac{{\hat{y}}_{i}-y_{i}}{y_{i}}\right|$$

where $y_{i}$ represents the true body weight of beef cattle, ${\hat{y}}_{i}$ represents the predicted body weight of beef cattle, $\bar{y}$ represents the average of the true body weights of beef cattle, and n represents the number of beef cattle samples.

## 3. Results

### 3.1. Comparison of the Performance of Different Instance Segmentation Models and the Effect of Instance Segmentation on Body Weight Estimation

In the task of dual-view beef cattle body weight estimation, instance segmentation of dual-view cattle images can effectively eliminate the interference of complex backgrounds. However, the segmentation process should preserve contour and texture information at the cattle boundaries as much as possible, so as to avoid prediction errors caused by the loss of edge details. In this paper, YOLOv8n-seg, YOLOv8s-seg, YOLO11s-seg, YOLO11n-seg, and EMA-YOLO11n-seg were compared in terms of Precision, Recall, mAP@0.5, mAP@0.5:0.95, Dice, Mask IoU, Params, and GFLOPs on the validation set to select the optimal instance segmentation model for dual-view beef cattle images.

As shown in Table 2, EMA-YOLO11n-seg achieved the best overall performance. For top-view images, it obtained the highest Precision, Recall, mAP@0.5, mAP@0.5:0.95, and Mask IoU, while maintaining a competitive Dice value. For side-view images, EMA-YOLO11n-seg achieved the highest Precision, mAP@0.5, Dice, and Mask IoU, and also maintained high Recall and mAP@0.5:0.95. In addition, its parameter size was equal to YOLO11n-seg and lower than the other three models, while its GFLOPs were the lowest among all models. Therefore, EMA-YOLO11n-seg was selected as the instance segmentation model for dual-view beef cattle images. Representative failure cases, mainly caused by severe occlusion, incomplete cattle body regions, or unclear boundaries, are provided in Appendix A.3.

To verify the effectiveness of instance segmentation in beef cattle body weight estimation, comparative experiments were conducted using a two-stream ResNet50 network on both the original dual-view RGB images and the segmented dual-view images generated by EMA-YOLO11n-seg. The experimental results are shown in Table 3. After instance segmentation, the MAE decreased by 2.35 kg, the RMSE decreased by 2.22 kg, and the R^2^ increased by 0.02. All evaluation metrics were improved compared with those obtained before segmentation, indicating that instance segmentation can effectively reduce the interference of irrelevant factors, such as complex backgrounds, and improve the regression accuracy of beef cattle body weight estimation.

### 3.2. Body Weight Estimation Results of Beef Cattle Using Different Feature Extraction Networks

In this study, several advanced deep neural networks were selected as alternative backbone networks for feature extraction, including EfficientNet, DenseNet, ResNet18, and ResNet50. For each backbone, a two-stream weight estimation network was constructed to separately learn dorsal and abdominal features of beef cattle and to perform dual-view feature fusion for body weight regression. Their performance was compared on the validation set to determine the optimal feature extraction network. The comparison results are shown in Table 4.

The proposed CBAM-ResNet50-SE feature extraction network achieved higher estimation accuracy for dual-view beef cattle body weight estimation than all other comparison methods on the validation set. Specifically, at the animal level, the MAE of CBAM-ResNet50-SE was 9.01 kg, which was reduced by 20.96 kg, 10.69 kg, 9.98 kg, and 2.36 kg compared with EfficientNet, DenseNet, ResNet18, and ResNet50, respectively. The RMSE was 14.34 kg, which was reduced by 25.44 kg, 13.67 kg, 11.38 kg, and 2.58 kg, respectively. The R^2^ value reached 0.92, which was higher than those of EfficientNet, DenseNet, ResNet18, and ResNet50 by 0.17, 0.18, 0.17, and 0.05, respectively.

In terms of model complexity, CBAM-ResNet50-SE had 52.12 M parameters, which were 6.87 M, 8.99 M, 28.67 M, and 1.82 M higher than those of EfficientNet, DenseNet, ResNet18, and ResNet50, respectively. Its computational cost was 9.21 GFLOPs, which was 1.56 GFLOPs, 3.23 GFLOPs, 5.24 GFLOPs, and 0.72 GFLOPs higher than those of the corresponding comparison networks. Overall, the results indicate that CBAM-ResNet50-SE improved the overall estimation performance, especially in terms of RMSE, with only a slight increase compared with the baseline ResNet50 network. The relatively larger model capacity helps enhance the representation of beef cattle body structure features, thereby improving the overall performance of dual-view beef cattle body weight estimation.

**Figure 5:**
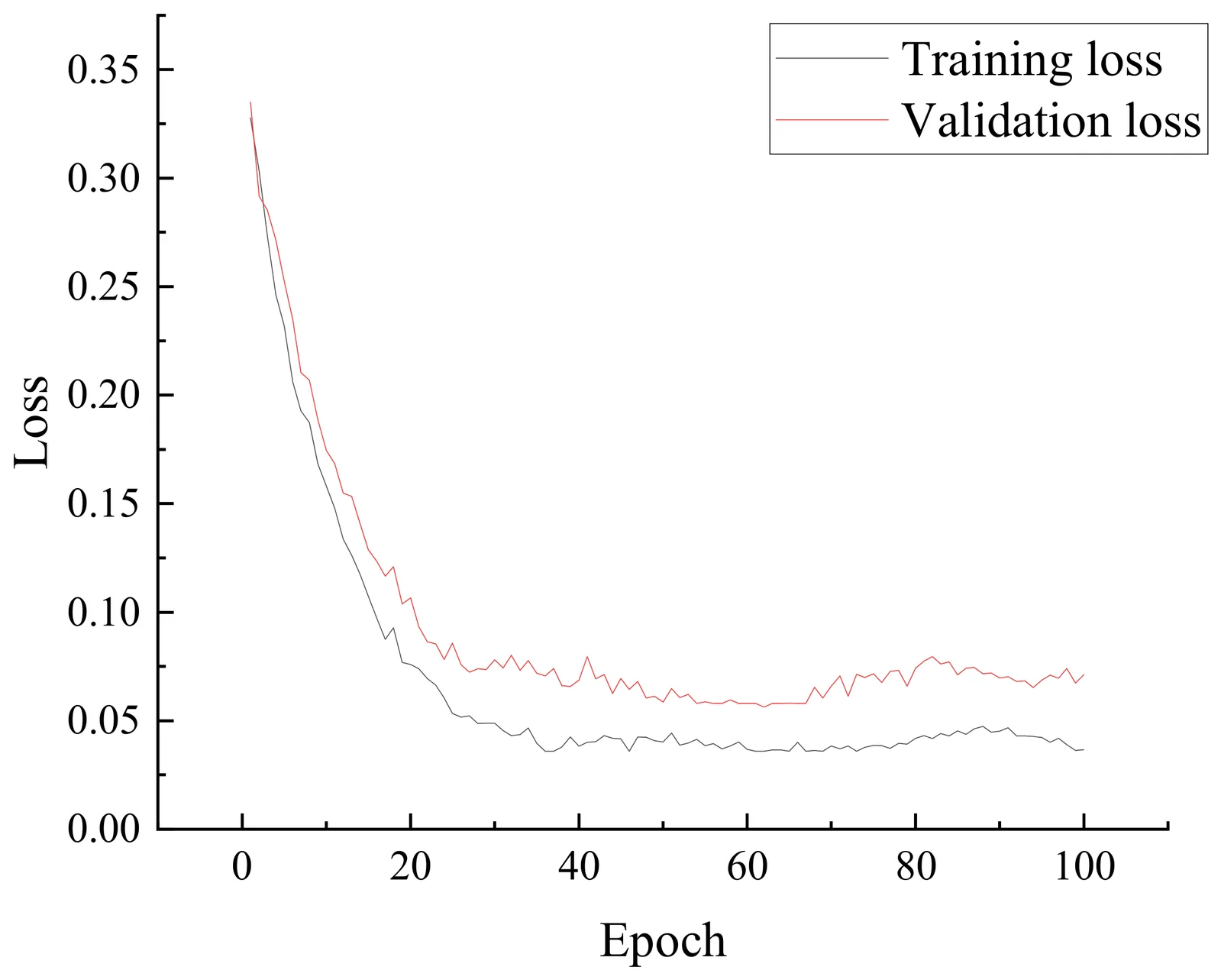
Training and validation loss curves of CBAM-ResNet50-SE.

The training and validation loss curves of the proposed CBAM-ResNet50-SE model are shown in Figure 5. Both losses decreased rapidly in the early stage and gradually stabilized, indicating good convergence; the checkpoint with the lowest validation loss was selected as the best model for final evaluation on the independent test set.

Because multiple image pairs from the same animal were not statistically independent, 95% confidence intervals were estimated using cattle-level clustered bootstrap resampling. For image-level evaluation, cattle identities were sampled with replacement in each bootstrap iteration, and all image pairs belonging to the selected cattle were retained to recalculate image-level MAE, RMSE, and R^2^. For animal-level evaluation, the predicted body weights of all image pairs from the same animal were first averaged, and the metrics were then recalculated based on the resampled cattle. This procedure was repeated 1000 times, and the 2.5th and 97.5th percentiles were used as the 95% confidence intervals. As shown in Table 5, the proposed model achieved image-level MAE, RMSE, and R^2^ values of 14.96 kg, 17.86 kg, and 0.85, respectively, on 660 image pairs from 22 independent Simmental cattle. After animal-level aggregation, the MAE, RMSE, and R^2^ were 14.28 kg, 16.92 kg, and 0.86, respectively. These results suggest that the proposed model maintained stable body weight estimation performance when independent cattle were used as the evaluation unit.

To further examine whether the model performance depended on a single dataset split, stratified five-fold cross-validation was conducted at the animal level. Cattle identity was used as the splitting unit, and growth stage was used as the stratification factor to ensure that each fold contained cattle from all growth stages. The proposed model achieved animal-level MAE, RMSE, and R^2^ values of 14.95 ± 1.00 kg, 18.32 ± 1.63 kg, and 0.858 ± 0.004, respectively. Detailed fold distribution and per-fold results are provided in Appendix A Table A2 and Table A3.

### 3.3. Comparative Analysis of Beef Cattle Body Weight Estimation Results Under Different Views

Compared with a single view, dual-view images can provide more comprehensive contour and spatial information for the body weight regression network, thereby compensating for feature loss caused by viewpoint limitations under a single-view setting and improving body weight estimation accuracy. To analyze the advantage of the two-stream structure in dual-view beef cattle body weight regression, comparative experiments were conducted using a single-stream ResNet50 network based on top-view images, a single-stream ResNet50 network based on side-view images, and a two-stream ResNet50 network based on dual-view images. The results are shown in Table 6.

After using dual-view beef cattle images, the estimation accuracy was clearly improved compared with that obtained using single-view data. The dual-view network achieved an MAE of 15.18 kg, an RMSE of 21.54 kg, and an R^2^ of 0.83. Compared with the top-view single-stream network, the MAE decreased by 10.67 kg, the RMSE decreased by 12.13 kg, and the R^2^ increased by 0.12. Compared with the side-view single-stream network, the MAE decreased by 3.64 kg, the RMSE decreased by 3.21 kg, and the R^2^ increased by 0.08.

In terms of model complexity, because the dual-view method adopts a two-stream feature extraction network, its parameter count and computational cost were both increased. Nevertheless, the results indicate that dual-view images can provide more complete contour and spatial structure information of beef cattle. By fusing complementary features from the top view and side view, the proposed method can effectively reduce information loss and estimation errors under single-view conditions, thereby improving the model’s representation of key body-shape features and the accuracy of body weight estimation.

### 3.4. Ablation Study

An ablation study was conducted on the validation set to evaluate the effects of attention modules and feature refinement modules on beef cattle body weight estimation. To reduce the influence of randomness, each experiment was repeated three times with different random seeds, and the results are reported as mean ± standard deviation. As shown in Table 7, the two-stream ResNet50 baseline achieved image-level MAE and RMSE values of 13.59 ± 0.63 kg and 20.50 ± 0.55 kg, respectively. After introducing different attention modules, CBAM + ResNet50 achieved better overall performance than MCA [33] + ResNet50 and CA [34] + ResNet50, especially in terms of RMSE. Based on CBAM + ResNet50, SE further improved the dual-view feature refinement performance. The final CBAM + ResNet50 + SE model achieved the best results on the validation set, with image-level MAE and RMSE values of 11.91 ± 0.66 kg and 18.05 ± 0.23 kg, and animal-level MAE and RMSE values of 9.28 ± 0.29 kg and 14.34 ± 0.33 kg, respectively. Compared with the baseline, the final model improved estimation accuracy with only a slight increase in parameters and GFLOPs. Therefore, CBAM + ResNet50 + SE was selected as the final structure for dual-view beef cattle BW estimation based on the validation results.

### 3.5. Comparative Analysis of Beef Cattle Body Weight Estimation Results Under Different Models and Input Settings

**Figure 6:**
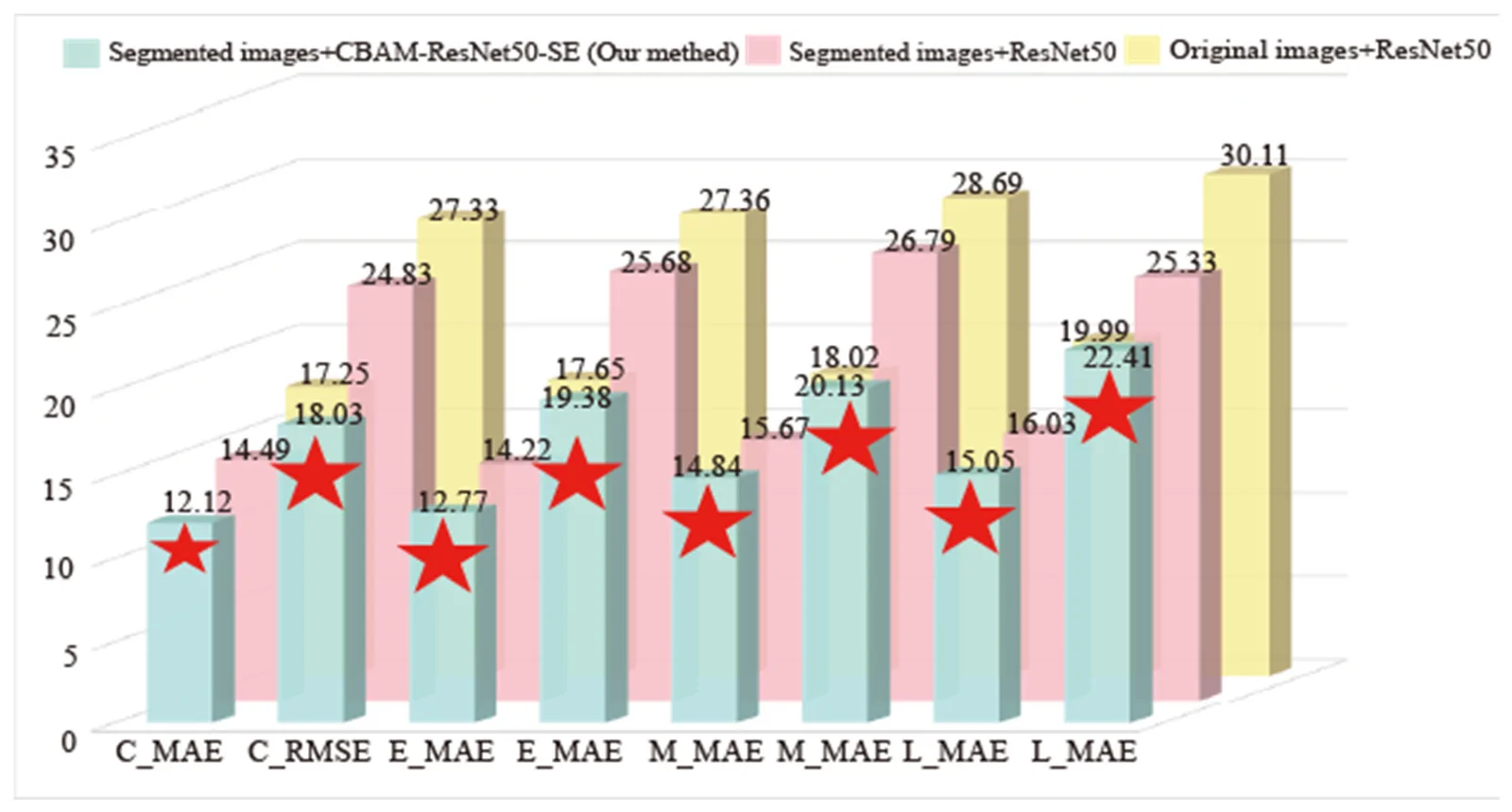
Comparison of body weight estimation performance under different input settings and network structures. Note: C, E, M, and L represent beef cattle in the calf stage, early fattening stage, middle fattening stage, and late fattening stage, respectively. MAE denotes mean absolute error and RMSE denotes root mean square error. Therefore, C_MAE, E_MAE, M_MAE, and L_MAE represent the MAE values for beef cattle at different growth stages, while C_RMSE, E_RMSE, M_RMSE, and L_RMSE represent the corresponding RMSE values (the red stars indicate the best-performing results).

To verify the effectiveness of image instance segmentation and the improved two-stream network in beef cattle body weight estimation, three groups of comparative experiments were designed in this study. First, the original dual-view beef cattle images were input into a two-stream body weight regression network using ResNet50 as the baseline model. Second, the segmented images generated by EMA-YOLO11n-seg were input into the same two-stream network for body weight estimation. Finally, the segmented images generated by EMA-YOLO11n-seg were input into a two-stream body weight regression model using CBAM-ResNet50-SE as the backbone network. The prediction results of different models on the test set are shown in Figure 6.

As shown in Figure 6, the segmented images combined with the CBAM-ResNet50-SE network achieved the best results on the test sets of beef cattle at different fattening stages. Compared with the unsegmented images and the original ResNet50 model, the improved network achieved an MAE of 12.12 kg and an RMSE of 18.03 kg for calves, corresponding to reductions of 29.74% and 34.03%, respectively, compared with the unsegmented images, and reductions of 16.36% and 27.39%, respectively, compared with the segmented images with the original ResNet50 model.

For beef cattle in the early fattening stage, the CBAM-ResNet50-SE network achieved an MAE of 12.77 kg, which was 27.65% and 10.20% lower than those obtained before segmentation and after segmentation with the original ResNet50, respectively. Its RMSE was 19.38 kg, representing reductions of 29.17% and 24.53%, respectively. For beef cattle in the middle fattening stage, the CBAM-ResNet50-SE network achieved an MAE of 14.84 kg and an RMSE of 20.13 kg, which were 17.65% and 29.84% lower than those obtained before segmentation and 5.30% and 24.86% lower than those obtained after segmentation with the original ResNet50, respectively. For beef cattle in the late fattening stage, the CBAM-ResNet50-SE network achieved an MAE of 15.05 kg and an RMSE of 22.41 kg, which were 24.71% and 25.57% lower than those of the original image + ResNet50 framework and 6.11% and 11.53% lower than those of the EMA-YOLO11n-seg + original ResNet50 method, respectively.

Overall, the proposed beef cattle body weight estimation method achieved favorable prediction performance on beef cattle datasets at different fattening stages, demonstrating its effectiveness for body weight estimation across different growth stages.

### 3.6. Analysis of Beef Cattle Body Weight Estimation Results Based on Dual-View RGB Images

**Figure 7:**
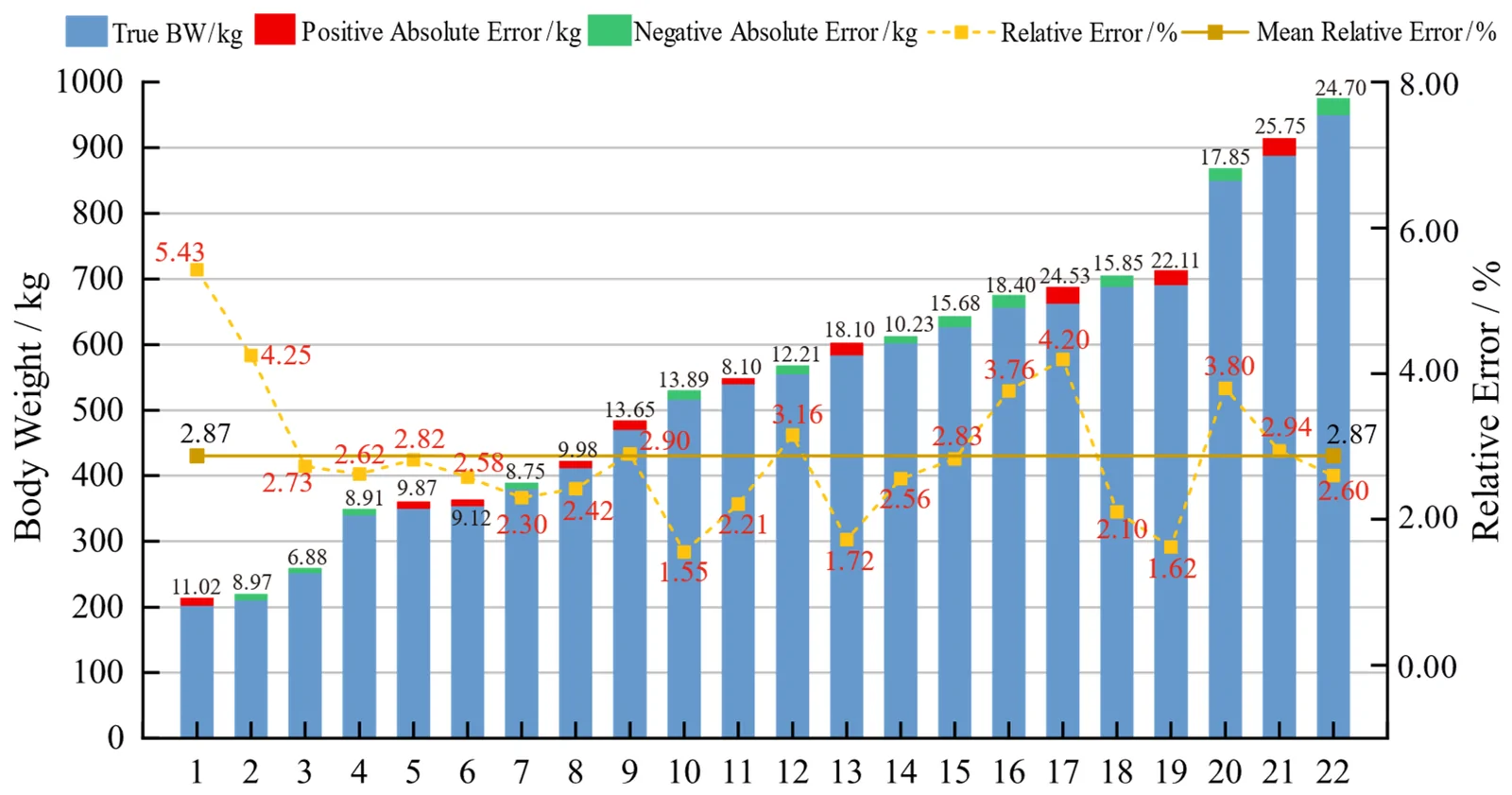
Body weight estimation results and error analysis for 22 beef cattle in the test set. (No. 1–4 were at the calf stage, No. 5–9 were at the early fattening stage, No. 10–16 were at the middle fattening stage, and No. 17–22 were at the late fattening stage).

The BW estimation results and error analysis of the proposed model for the 22 beef cattle in the test set are shown in Figure 7. In the figure, the blue bars represent the ground-truth body weights of the cattle, whereas the red or green bars indicate the absolute errors between the estimated values and the true values. Red bars indicate overestimation, while green bars indicate underestimation. As shown in Figure 7, the relative estimation error for all individual cattle was controlled within 6%, with a mean relative error of 2.83%. These results indicate that the proposed method has a strong learning capability, enabling it to effectively capture information from both dorsal and lateral body-shape information of beef cattle and dynamically adjust model weights, thereby improving body weight estimation accuracy.

### 3.7. Analysis of Beef Cattle Body Weight Estimation Results Under Different Postures

**Figure 8:**
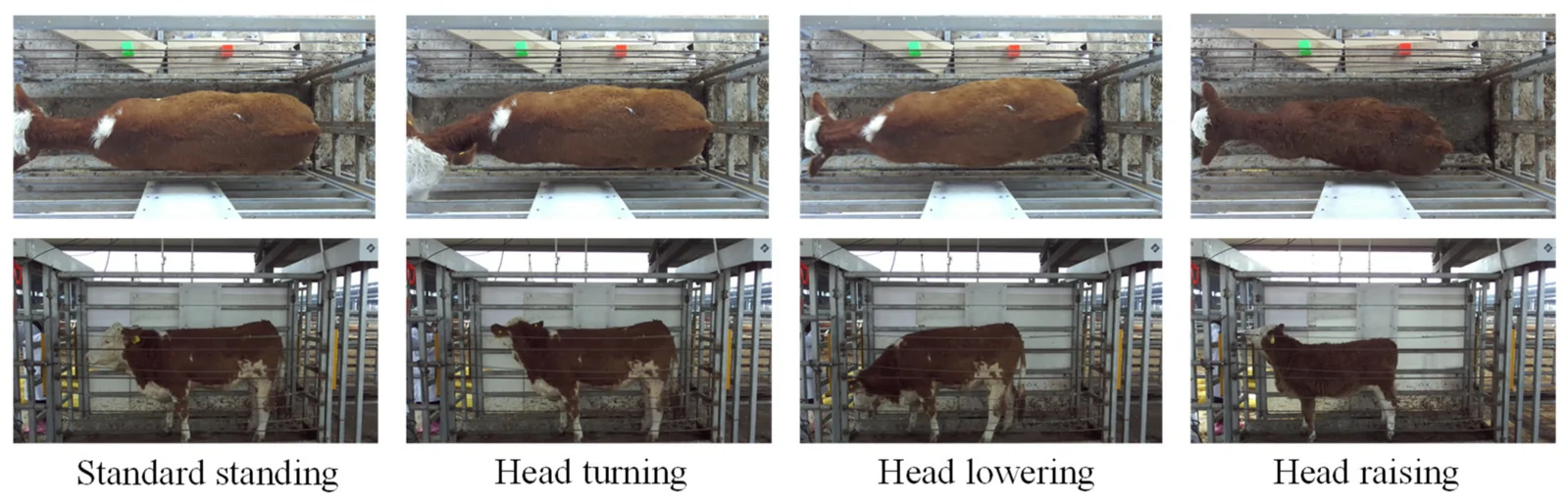
Representative dual-view images of beef cattle under different postures.

During the weighing process, beef cattle often exhibit different natural postures, such as head turning, head raising, head lowering, and standard standing. These posture variations may lead to changes in body contour and local morphological features, which may in turn affect body weight estimation results. To evaluate the adaptability and estimation stability of the proposed BW estimation model under different posture conditions, four representative posture categories were selected for comparative analysis. The posture category of each image pair was jointly classified by three experts with more than three years of experience in beef cattle production or related research. Representative dual-view images of these postures are shown in Figure 8.

The BW estimation results under different posture conditions are presented in Table 8. The results show that the proposed method maintained good estimation performance under all posture conditions, with the MAE ranging from 12.68 kg to 16.46 kg and the RMSE ranging from 14.89 kg to 21.37 kg, indicating that the proposed method has a certain degree of adaptability to posture variation in beef cattle. Specifically, the model achieved the best performance under the standard standing posture, with an MAE of 12.68 kg and an RMSE of 14.89 kg. In contrast, the largest errors were observed under the head-lowering posture, where the MAE and RMSE reached 16.46 kg and 21.37 kg, respectively, suggesting that head lowering may affect the complete representation of body-shape features to some extent. By comparison, the estimation results under the head-raising and head-turning postures still maintained relatively high accuracy. Under the head-raising posture, the MAE and RMSE were 13.77 kg and 16.74 kg, respectively, whereas under the head-turning posture, they were 14.32 kg and 18.22 kg, both of which were better than those obtained under the head-lowering condition.

### 3.8. Validation on the Sanhe Cattle Dataset

To further evaluate the adaptability of the proposed framework to another beef cattle breed, Sanhe cattle data collected in October 2023 in Hulunbuir, Inner Mongolia, were used for additional validation in this study. This dataset contained 68 Sanhe cattle, and 10 pairs of dual-view images were collected for each animal, resulting in a total of 680 dual-view image pairs. The body weight of the 68 Sanhe cattle ranged from 274 to 515.5 kg, including four cattle at the calf stage, 52 at the early fattening stage, and 12 at the middle fattening stage.

**Figure 9:**
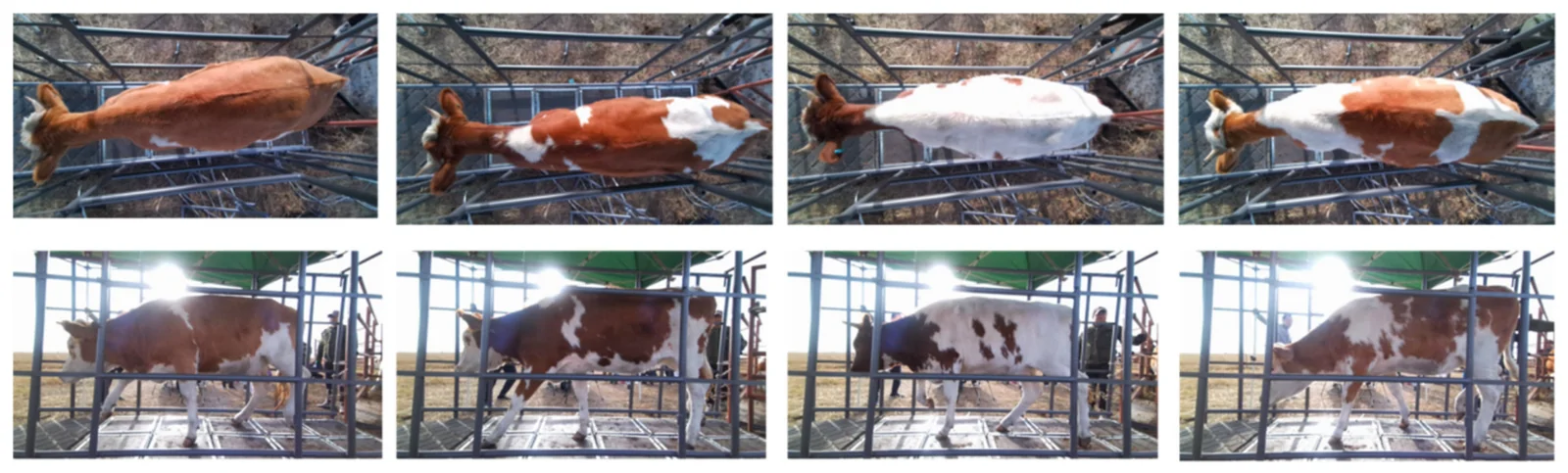
Representative dual-view images of Sanhe cattle.

Representative dual-view images of the collected Sanhe cattle are shown in Figure 9. First, the EMA-YOLO11n-seg network was used to segment the multi-view beef cattle images, yielding 680 segmented dual-view image pairs of Sanhe cattle. For the adaptation experiments, the Sanhe cattle dataset was divided at the animal level into a training set, a validation set, and an independent test set. Specifically, 44 cattle with 440 image pairs were used as the training set, including two calves, 34 early-fattening cattle, and eight middle-fattening cattle. Another 10 cattle with 100 image pairs were used as the validation set, including one calf, eight early-fattening cattle, and one middle-fattening cattle. The remaining 14 cattle with 140 image pairs were used as the independent test set for final performance evaluation, including one calf, 10 early-fattening cattle, and three middle-fattening cattle. Data augmentation was applied only to the training set, resulting in 1320 dual-view image pairs for model training. The validation and test sets were not augmented.

To distinguish direct cross-breed generalization from target-breed adaptation, three validation settings were designed using the same two-stream CBAM-ResNet50-SE body weight estimation model. First, in the zero-shot cross-breed validation, the CBAM-ResNet50-SE model trained on the Simmental cattle dataset was directly applied to the entire Sanhe cattle dataset without using any Sanhe samples for training, validation, fine-tuning, or parameter adjustment. Second, in the fine-tuning validation, the Simmental-trained CBAM-ResNet50-SE model was used as the initial model and further fine-tuned using the Sanhe training subset, with the best checkpoint selected according to the Sanhe validation-set performance. Third, in the breed-specific retraining validation, the two-stream CBAM-ResNet50-SE model was trained using the Sanhe training subset, and the best checkpoint was selected based on the Sanhe validation set. The fine-tuning and breed-specific retraining models were finally evaluated on the same independent Sanhe test set. The comparison results are shown in Table 9.

**Figure 10:**
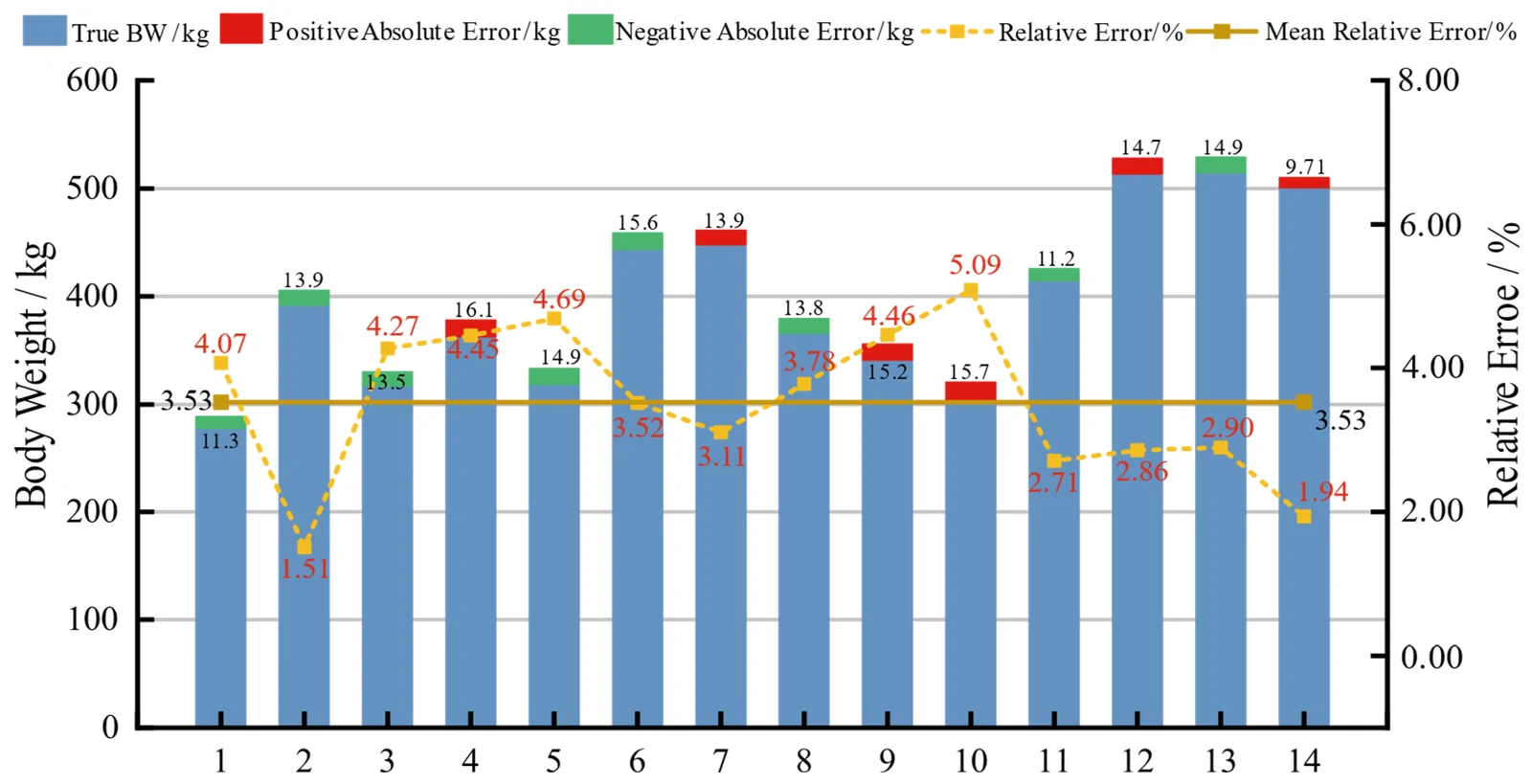
Body weight estimation results and error analysis for 14 Sanhe cattle in the validation set. (No. 1 was at the calf stage, No. 2–11 were at the early fattening stage, and No. 12–14 were at the middle fattening stage.).

As shown in Table 9, under the zero-shot cross-breed validation setting, the model trained on the Simmental cattle dataset achieved animal-level MAE, RMSE, and R^2^ values of 30.50 kg, 37.98 kg, and 0.59, respectively, when directly applied to the entire Sanhe cattle dataset. This indicates that the model retained a certain cross-breed prediction ability without using any Sanhe samples for training or fine-tuning, although differences in body shape, coat pattern, and body contour among breeds affected its direct generalization performance. After fine-tuning with the Sanhe training subset, the animal-level MAE on the independent test set decreased to 19.59 kg, and the R^2^ increased to 0.82. Under the breed-specific retraining setting, the model achieved the best performance, with animal-level MAE, RMSE, and R^2^ values of 16.90 kg, 20.16 kg, and 0.87, respectively. The BW prediction results for the 14 Sanhe cattle in the independent test set are shown in Figure 10, with an MRE of 3.53%.

## 4. Discussion

This paper proposed a non-contact beef cattle weight estimation method based on instance segmentation and dual-view feature fusion. The method combines the EMA-YOLO11n-seg instance segmentation network with the CBAM-ResNet50-SE two-stream regression network, enabling the model to automatically extract cattle morphological features related to body weight in natural farming environments. The experimental results showed that instance segmentation, dual-view fusion, and attention mechanisms all contributed positively to improving weight estimation accuracy. The proposed method not only reduced the influence of background interference on the regression model, but also improved its adaptability to beef cattle at different fattening stages and under different postures, and showed potential adaptability on the Sanhe cattle dataset.

Instance segmentation has been proven beneficial for beef cattle weight estimation [21,22], and the present study further confirmed this finding. The experimental results showed that EMA-YOLO11n-seg effectively removed complex background interference. Compared with directly using original dual-view RGB images, the segmented images reduced the MAE from 17.53 kg to 15.18 kg and the RMSE from 23.76 kg to 21.54 kg, indicating that background removal and cattle body region extraction play an important role in image-based weight estimation.

This paper showed that, compared with single-view RGB images, dual-view or multi-view RGB image fusion performed better than single-view input in cattle body weight or weight-related trait estimation, which is consistent with the findings of [35,36]. Specifically, top-view and side-view images provide complementary body-shape information. The top view mainly reflects dorsal contour, body length, and body width, whereas the side view supplements body height, abdominal contour, and body depth. A single view is prone to information loss due to view limitation, partial occlusion, or posture variation, whereas dual-view fusion can supplement cattle structural information from different directions and provide a more complete body-shape representation. The experimental results verified this point, as shown in Table 6, indicating that there is clear information complementarity between the top and side views. The two-stream structure can effectively fuse body-shape features from different views, thereby improving weight estimation performance. On the basis of dual-view fusion, the attention mechanisms further enhanced the model’s ability to represent key body-shape features. Beef cattle body weight is closely related to features such as dorsal width, trunk area, abdominal contour, and body depth, but not all image regions and channels contribute equally to weight estimation. The CBAM module reweights features from both spatial and channel dimensions, allowing the network to focus more on key regions related to body weight. The SE module further recalibrates channel weights after dual-view feature fusion, enhancing effective features and suppressing redundant information. Therefore, the combination of CBAM and SE helps improve the discriminative ability of fused features and enables the model to make better use of dual-view body-shape information. The ablation study further confirmed the effectiveness of the attention mechanisms, as shown in Table 7. With the combination of these two modules, the model could more fully learn morphological features related to body weight, achieving more stable and accurate beef cattle weight estimation.

The results for different growth stages showed that the proposed method can be applied to beef cattle at different fattening stages, although the estimation difficulty varied among stages. This is consistent with previous computer vision-based beef cattle weight estimation studies [8,19]. In the early growth stage, cattle body shape changes rapidly, individual differences are relatively large, and the mapping relationship between image features and body weight is less stable; therefore, estimation errors are more likely to be affected by individual variation. As cattle enter the middle and late fattening stages, body structure gradually stabilizes, muscle and fat deposition patterns become clearer, and visual features related to body weight become more stable [37,38]. This may explain why the model maintained good performance across different stages while still showing stage-specific error differences.

Posture variation remains an important factor affecting image-based weight estimation. Previous studies have shown that animal posture changes can influence the stability of visual weight estimation and non-contact body size measurement [39,40]. The results of this study showed that the model maintained a certain estimation ability under head-raising, head-lowering, head-turning, and standard standing postures. Among them, the best performance was obtained under the standard standing posture, whereas relatively larger errors were observed under the head-lowering posture. This may be because head lowering changes the local body contour, causes occlusion of key regions, or deforms the side-view body structure. These results indicate that the proposed method has a certain degree of posture adaptability, but posture variation remains an important factor affecting the stability of image-based weight estimation. In practical applications, the introduction of posture recognition, posture correction, or multi-frame image selection mechanisms may further improve estimation stability.

The results obtained on the Sanhe cattle dataset further suggest that the proposed dual-view feature fusion framework has potential adaptability to another beef cattle breed. The model achieved an MAE of 17.56 kg and an RMSE of 20.71 kg on the Sanhe validation set, with an individual-level mean relative error of 3.53%. Since beef cattle of different breeds may differ in body structure, fat distribution, and growth patterns, these results indicate that dual-view morphological features can be adapted to cattle with different breed characteristics. Further independent validation without breed-specific training samples is still needed to rigorously evaluate cross-breed generalization.

To further evaluate the application value of the proposed method, this study compared it with several advanced animal weight estimation studies in terms of backbone architecture, animal species, input data type, imaging view, and estimation accuracy, as shown in Table 10.

Zhang et al. (2025) [36] achieved an MAE of 13.814 kg for carcass weight estimation using data from 2023 beef cattle, which was lower than that obtained in the present study. Compared with animal body weight, carcass weight is less affected by feed intake, water intake, gastrointestinal contents, and natural posture variation, and it usually has a more direct correspondence with the body contour and tissue structure observable in images. Therefore, lower prediction errors are more readily achieved. Liu et al. (2023) [35] achieved a MAPE of 4.1% using multi-view RGB videos of 196 dairy cows. Although this approach can provide richer body-shape information, its camera arrangement and system implementation are relatively complex, which may be less favorable for long-term equipment maintenance. Compared with [41], who estimated yak body weight from single side-view RGB images by first estimating body size traits and then regressing body weight from these traits, the proposed method avoids a two-stage estimation process in which errors may accumulate. In addition, single side-view input provides relatively limited body-shape information, especially in representing dorsal contour, body width, and overall trunk structure. Compared with [42], who used side-view RGB-D images for sheep weight estimation, the sheep body weight range was relatively small, from 19.5 to 94 kg. Overall, the proposed method achieves stable automatic estimation in a relatively wide live body weight range using only dual-view RGB images. By combining instance segmentation with an attention-enhanced two-stream network, the method balances body-shape information completeness, deployment convenience, and estimation performance, achieving a favorable trade-off among accuracy, cost, and engineering applicability.

However, this study still has several limitations. First, the main dataset was collected from limited farming scenarios, and the diversity of environmental backgrounds, lighting conditions, breeds, and sexes still needs to be expanded. Second, the experiments were conducted in a shaded, fixed weighing-channel scenario with fixed dual-view cameras; therefore, the current results mainly support application under fixed weighing-channel conditions, and further validation is needed before extending the method to open barns, severe occlusion, or unconstrained free-walking scenarios. Third, although RGB images are low-cost and easy to deploy, they lack direct three-dimensional depth information and are susceptible to shadows, surface texture, and partial occlusion. Future research could further integrate RGB images, depth images, video temporal information, or posture information, and conduct lightweight deployment and real-time inference tests.

## 5. Conclusions

This paper addresses the incomplete body-shape representation of single-view RGB images and the interference of complex backgrounds in beef cattle weight estimation by proposing a beef cattle weight estimation method based on dual-view RGB images. The proposed method first uses EMA-YOLO11n-seg to perform instance segmentation on top-view and side-view images, thereby reducing the influence of complex backgrounds on subsequent estimation. Then, a two-stream CBAM-ResNet50-SE network is constructed to extract dorsal and lateral body-shape features of beef cattle separately, and weight regression is achieved through attention-enhanced feature fusion. The experimental results show that EMA-YOLO11n-seg performs well in the dual-view beef cattle image segmentation task, with mAP@0.5 values of 99.18% and 98.35% for top-view and side-view images, respectively. In the weight estimation task, CBAM-ResNet50-SE achieves the best performance, with an MAE, RMSE, and R^2^ of 14.96 kg, 17.86 kg, and 0.85, respectively. Further analysis shows that the proposed method maintains stable estimation performance across cattle at different growth stages and under different posture conditions, and shows potential adaptability on the Sanhe cattle dataset. In summary, the proposed method provides a feasible solution for non-contact beef cattle weight estimation in fixed weighing-channel scenarios, but further validation is needed before applying it to open barns, severe occlusion, or unconstrained free-walking conditions.

## Figures and Tables

**Table 1:** Characteristics and data distribution of the beef cattle dataset across different growth stages.

Stage	Num	Weight Range/kg	Mean ± SD/kg	Number of Cattle			RGB Image Pairs		
				Train	Val	Test	Train	Val	Test
Calves	21	169–300	233.41 ± 39.01	14	3	4	420	90	120
Early	28	319–439	376.77 ± 30.40	18	5	5	540	150	150
Middle	31	453–648	554.94 ± 53.17	19	5	7	570	150	210
Late	27	656–980	773.13 ± 97.27	17	4	6	510	120	180
All	107	169–980	489.52 ± 217.11	68	17	22	2040	510	660

**Table 2:** Comparison of instance segmentation performance across models for multi-view images of beef cattle.

Top view								
Model	Precision/%	Recall/%	mAP@0.5/%	mAP@0.5:0.95/%	Dice/%	Mask IoU/%	Parameters/M	GFLOPs
YOLOv8n-seg	85.63	84.53	89.65	86.45	92.61	92.33	3.4	12.6
YOLOv8s-seg	89.64	85.46	92.52	88.98	95.14	94.90	11.8	42.6
YOLO11s-seg	97.36	95.65	98.54	95.31	98.82	97.33	10.1	35.5
YOLO11n-seg	97.24	91.45	97.68	90.81	96.59	93.51	2.9	10.2
EMA-YOLO11n-seg	99.66	95.82	99.18	98.78	98.75	97.54	2.9	9.1
Side view								
Model	Precision/%	Recall/%	mAP@0.5/%	mAP@0.5:0.95/%	Dice/%	Mask IoU/%	Parameters/M	GFLOPs
YOLOv8n-seg	81.64	80.33	89.12	84.20	88.27	87.61	3.4	12.6
YOLOv8s-seg	84.75	84.56	92.32	88.14	94.43	94.66	11.8	42.6
YOLO11s-seg	97.26	95.11	98.01	95.88	97.45	95.29	10.1	35.5
YOLO11n-seg	97.01	87.35	96.33	94.03	98.04	96.17	2.9	10.2
EMA-YOLO11n-seg	98.65	94.10	98.35	96.67	98.13	96.34	2.9	9.1

**Table 3:** Effect of instance segmentation on beef cattle body weight estimation.

Model	MAE/kg	RMSE/kg	R^2^
Before Segmentation	17.53	23.76	0.81
EMA-YOLO11n-seg	15.18	21.54	0.83

**Table 4:** Comparison of different feature extraction networks for body weight estimation on the validation set.

Model	MAE/kg		RMSE/kg		R^2^		Params/M	GFLOPs
	Image-Level	Animal-Level	Image-Level	Animal-Level	Image-Level	Animal-Level		
EfficientNet [30]	35.64	29.97	47.78	39.78	0.73	0.75	45.25	7.65
DenseNet [31]	22.40	19.70	31.69	28.01	0.73	0.74	43.13	5.98
ResNet18 [32]	20.04	18.99	27.21	25.72	0.74	0.75	23.45	3.97
ResNet50 [32]	13.19	11.37	20.20	16.92	0.85	0.87	50.30	8.49
CBAM-ResNet50	12.84	11.16	18.17	17.55	0.89	0.90	51.97	9.13
CBAM-ResNet50-SE	11.98	9.01	17.96	14.34	0.90	0.92	52.12	9.21

**Table 5:** Image-level and animal-level performance of the proposed model on the independent Simmental test set.

Evaluation Level	No. of Independent Cattle	No. of Image Pairs	MAE/kg	95% CI	RMSE/kg	95% CI	R^2^	95% CI	MRE/%
Image-level	22	660	14.96	13.78–16.21	17.86	16.42–19.35	0.85	0.76–0.87	-
Animal-level	22	-	14.28	11.32–17.65	16.92	13.44–21.36	0.86	0.76–0.88	2.87

Note: Animal-level metrics were calculated by averaging all image-pair predictions from the same animal. The 95% CIs were estimated using cattle-level clustered bootstrap resampling with 1000 repetitions.

**Table 6:** Comparison of ResNet50-based body weight regression results under different views.

Input View	MAE/kg	RMSE/kg	R^2^	Params/M	GFLOPs
Top-view	25.85	33.67	0.71	25.63	4.37
Side-view	18.82	24.75	0.75	25.63	4.37
Dual-view	15.18	21.54	0.83	50.30	8.49

**Table 7:** Ablation study of different modules on the validation set.

Model	MAE/kg		RMSE/kg		Params/M	GFLOPs
	Image-Level	Animal-Level	Image-Level	Animal-Level		
ResNet50	13.59 ± 0.63	11.61 ± 0.39	20.50 ± 0.55	17.26 ± 0.51	50.30	8.49
MCA + ResNet50	13.83 ± 0.44	11.51 ± 0.35	19.29 ± 0.42	16.96 ± 0.41	50.94	8.91
CA + ResNet50	14.69 ± 0.27	12.36 ± 0.38	19.89 ± 0.42	17.45 ± 0.44	50.35	9.04
CBAM + ResNet50	13.20 ± 0.38	11.36 ± 0.28	18.75 ± 0.53	16.83 ± 0.66	51.97	9.13
CBAM + ResNet50 + MCA	12.76 ± 0.72	10.06 ± 0.34	18.30 ± 0.15	15.11 ± 0.17	53.45	9.43
CBAM + ResNet50 + SE	11.91 ± 0.66	9.28 ± 0.29	18.05 ± 0.23	14.34 ± 0.33	52.12	9.21

**Table 8:** Body weight estimation results under different postures.

Posture	Image Pairs	MAE/kg	RMSE/kg
Head raising	83	13.77	16.74
Head lowering	72	16.46	21.37
Head turning	109	14.32	18.22
Standard standing	396	12.68	14.89

**Table 9:** Comparison of zero-shot validation, fine-tuning validation, and breed-specific retraining validation on the Sanhe cattle dataset.

Experiment	Initial Weights	Training Strategy	Test Set	No. of Test Cattle	MAE/kg		RMSE/kg		R^2^	
					Image-Level	Animal-Level	Image-Level	Animal-Level	Image-Level	Animal-Level
Zero-shot cross-breed validation	Simmental-trained model	Trained on Simmental cattle without Sanhe fine-tuning	Entire Sanhe dataset	68	29.87	30.50	37.33	37.98	0.60	0.59
Fine-tuning validation	Simmental-trained model	Fine-tuned using the Sanhe training subset	Sanhe test set	14	20.96	19.59	25.76	24.17	0.81	0.82
Breed-specific retraining validation	Pretrained ResNet50 backbone	Trained the dual-stream CBAM-ResNet50-SE model using the Sanhe training subset	Sanhe test set	14	17.56	16.90	20.71	20.16	0.87	0.87

**Table 10:** Comparison with existing vision-based body weight estimation methods.

Model	Animal	Number	BW/kg	Input	View	Accuracy
Dual-input neural network [36]	Beef cattle	2023	151–563	RGB	Top view + side view	MAE = 13.814 kg
Multi-view video analytics [35]	Cow	196	458–876	RGB video	Multi-view	MAPE = 4.1%
Image analysis + regression model [41]	Yak	146	52.5–308.0	RGB	Side view	RMSE = 7.52–11.3 kg
LiteHRNet [42]	Sheep	726	19.5–94	RGB-D	Side view	MAE = 6.736 kg
EMA-YOLO11n-seg + CBAM-ResNet50-SE (ours)	Beef cattle	107	169–980	RGB	Top view + side view	MAE = 14.96 kg

**Figure A1:**
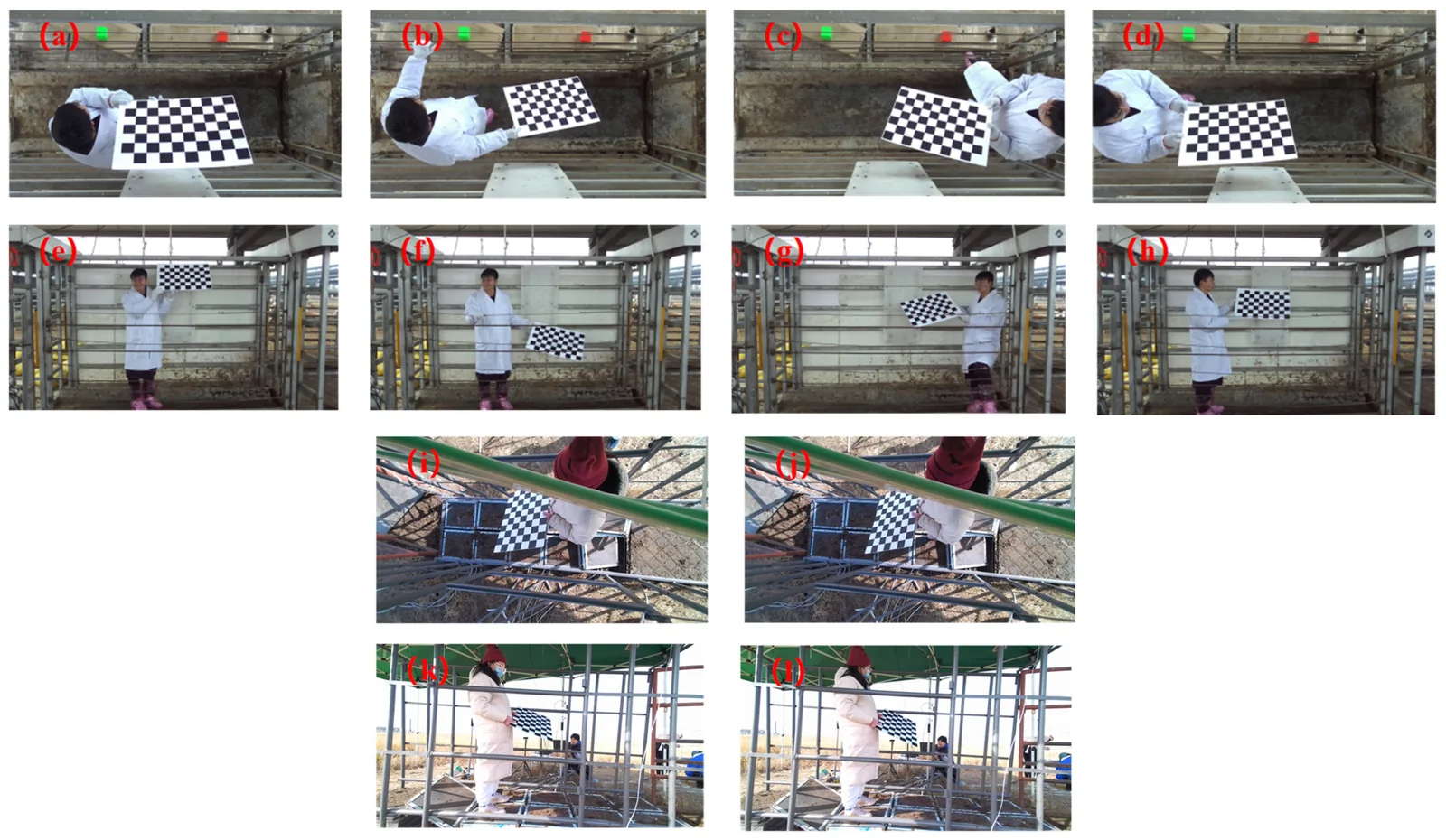
Example checkerboard calibration images of the dual-view RGB acquisition system. (**a**–**d**) Top-view camera calibration images and (**e**–**h**) side-view camera calibration images of the ZED 2i system used for Simmental cattle data acquisition; (**i**–**l**) representative checkerboard calibration images of the Microsoft Azure Kinect DK cameras used for Sanhe cattle data acquisition. Checkerboards with known physical dimensions (60 mm × 60 mm squares) were used for independent calibration of both acquisition systems, and images with different board positions and poses were used for camera calibration and image scale correction.

**Figure A2:**
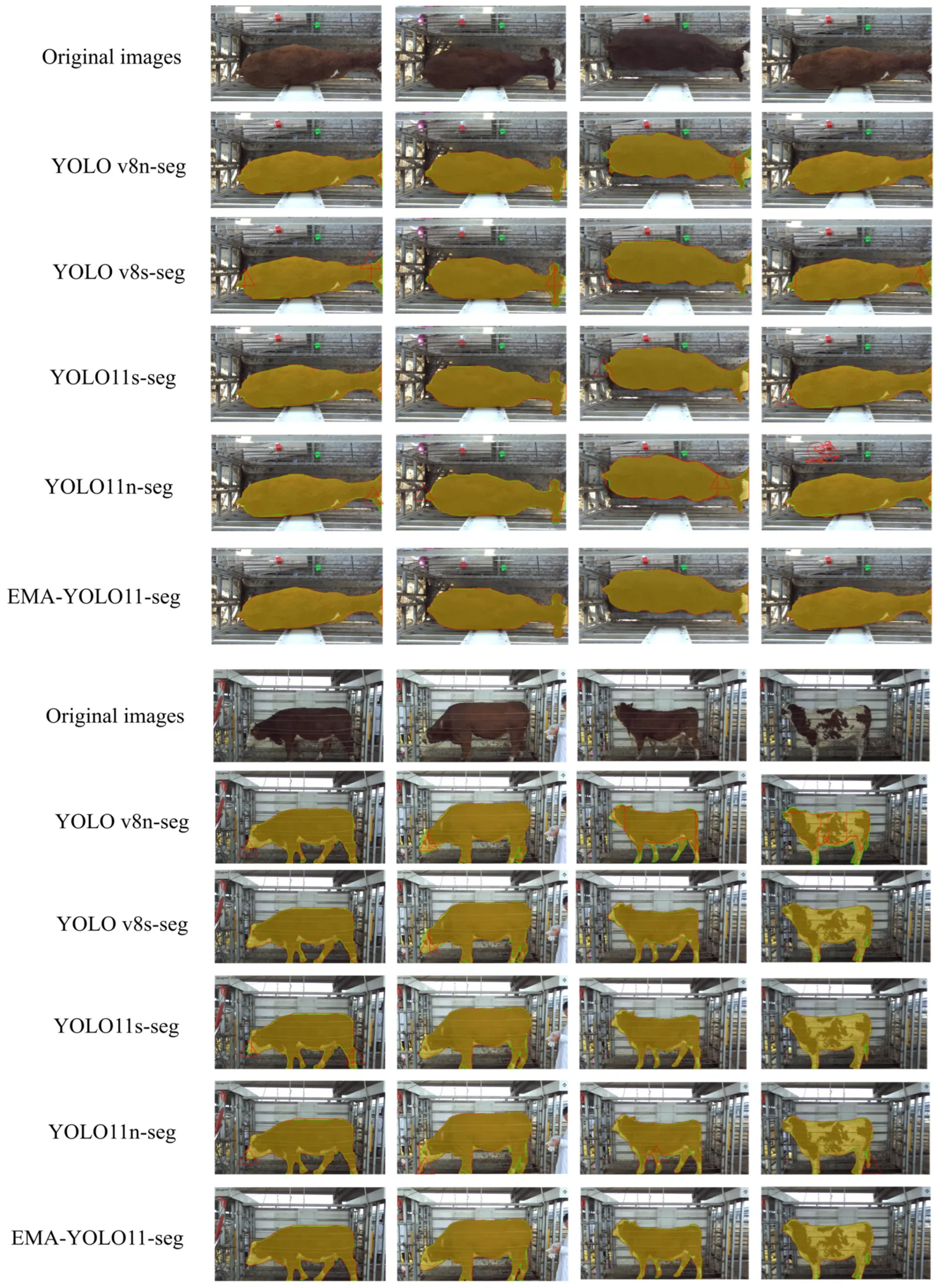
Visualization of beef cattle segmentation results and typical failure cases from top-view and side-view perspectives. The first row shows the original images, while the remaining rows show the segmentation results. The yellow regions indicate the predicted cattle body masks. The red and green contours represent the predicted boundaries and manually annotated boundaries, respectively. The red triangles and circles highlight typical under-segmentation, missed segmentation, and false segmentation regions.

## Data Availability

The data supporting the findings of this study are not publicly available at present because the dataset is still being used for ongoing related studies. The dataset will be made publicly available in an appropriate repository after all manuscripts based on these data have been published. Until then, the data may be made available from the corresponding author upon reasonable request, subject to institutional and ethical considerations. The source code used in this study is publicly available at: https://github.com/ziruoli425-svg/Beef-Cattle-Body-Weight-Estimation-based-on-Dual-View-RGB-Images.

## Abbreviations

The following abbreviations are used in this manuscript:

EMA	Efficient Multi-Scale Attention
CBAM	Convolutional Block Attention Module
SE	Squeeze-and-Excitation
P	Precision
R	Recall
mAP@0.5	Mean Average Precision at an IoU Threshold of 0.5
MAE	Mean Absolute Error
RMSE	Root Mean Square Error
MAPE	Mean Absolute Percentage Error
R2	R-Squared
RER	Relative Error Rate
MRE	Mean Relative Error

## Appendix A

### Appendix A.1. Feeding Management and Growth-Stage Classification of Experimental Cattle

The experimental cattle used in this study were Simmental beef cattle raised on a commercial farm. A total of 107 beef cattle were collected in the overall experiment, with a body weight range of 169–980 kg. Referring to the Beef Cattle Feeding Standard issued by the Ministry of Agriculture and Rural Affairs in 2004 [43] and the Technical Guidance on Quality and Safety Control of Beef Cattle issued in 2023 (Ministry of Agriculture and Rural Affairs, 2023) [44], and in combination with the actual feeding management practices of the experimental farm as well as the age and body weight distribution of the sampled cattle, the cattle were divided into four growth stages, namely the calf stage, early fattening stage, middle fattening stage, and late fattening stage.

**Table A1:** Growth-stage classification criteria for experimental beef cattle.

Growth Stage	Age Range/Months	Body Weight Range/kg
Calf stage	0~6	<300
Early fattening stage	7~12	300–450
Middle fattening stage	13~17	450–650
Late fattening stage	>18	> 650

Note: The growth-stage classification was determined with reference to the Beef Cattle Feeding Standard [43] and the Technical Guidance on Quality and Safety Control of Beef Cattle [44], together with the actual feeding management practices of the experimental farm and the age and body weight distribution of the sampled cattle.

### Appendix A.2. Camera Calibration and Reference-Plane Scale Normalization

To reduce imaging-scale differences caused by different camera installation positions and shooting distances, the top-view and side-view cameras of each acquisition system were calibrated independently. Both the ZED 2i cameras used for Simmental cattle and the Microsoft Azure Kinect DK cameras used for Sanhe cattle were independently calibrated using the same checkerboard with known physical dimensions (60 mm × 60 mm squares). Calibration images were collected at different positions and poses within the camera field of view, and representative checkerboard images are shown in Figure A1.

Based on the pixel coordinates of checkerboard corners and their corresponding physical coordinates, the camera intrinsic matrix, distortion coefficients, and the pose of the checkerboard relative to the camera were calculated. The camera imaging model is expressed as:

$$s{\left[u,v,1\right]}^{T}=K\left[R|t\right]{\left[X,Y,Z,1\right]}^{T}$$

where *X*, *Y*, and *Z* denote the physical coordinates of checkerboard corners in the world coordinate system; *u* and *v* denote the corresponding image pixel coordinates; *s* is the scale factor; *K* is the camera intrinsic matrix; and *R* and *t* are the rotation matrix and translation vector, respectively. The intrinsic matrix *K* is expressed as:

$$K=\left(\begin{array}{ccc} fx & 0 & cx\\ 0 & fy & cy\\ 0 & 0 & 1 \end{array}\right)$$

where fx and fy are the focal lengths in pixel units, and cx and cy are the principal point coordinates. The intrinsic parameters and distortion coefficients were estimated from the pixel and physical coordinates of the checkerboard corners, and the original RGB images were then corrected for distortion.

After camera intrinsic calibration, reference-plane scale normalization was further performed. For the top-view images, checkerboard images located near the main imaging region of the cattle back were selected to establish a horizontal reference plane close to the back plane. For the side-view images, checkerboard images located near the main side-body imaging region were selected to establish a vertical reference plane close to the cattle side plane. Based on the image coordinates of checkerboard corners and their real physical coordinates, homography matrices were calculated for the top-view and side-view images, respectively. The relationship between image coordinates and physical coordinates on the reference plane is expressed as:

$$\lambda {\left[Xp,Yp,1\right]}^{T}=H{\left[u^{\prime },v^{\prime },1\right]}^{T}$$

where *u*′ and *v*′ are the distortion-corrected pixel coordinates; *Xp* and *Yp* are the physical coordinates on the reference plane; *H* is the homography matrix; and λ is the scale factor. If the target normalized scale is set to *r* cm/pixel, the physical coordinates on the reference plane can be further converted into standardized image coordinates:

us=Xp/r,vs=Yp/r

where *us* and *vs* are the scale-normalized pixel coordinates. Through this process, the top-view and side-view images were mapped to standard image planes with a unified physical scale, reducing the influence of different camera installation conditions and imaging-scale differences on body weight regression. It should be noted that RGB-based planar scale normalization mainly reduces imaging-scale differences under fixed acquisition conditions; it cannot fully recover the three-dimensional body structure or completely eliminate errors caused by variations in cattle body height.

### Appendix A.4. Distribution of Cattle Across Growth Stages in Stratified Five-Fold Cross-Validation

**Table A2:** Distribution of cattle across growth stages in stratified five-fold cross-validation.

Fold	Calves	Early Fattening	Middle Fattening	Late Fattening	Total Cattle	Image Pairs
Fold 1 (main)	4	5	7	6	22	660
Fold 2	4	6	6	5	21	630
Fold 3	4	6	6	5	21	630
Fold 4	4	6	6	5	21	630
Fold 5	5	5	6	6	22	660

**Table A3:** Animal-level results of stratified five-fold cross-validation.

Fold	No. of Validation Cattle	No. of Validation Image Pairs	MAE/kg	RMSE/kg	R^2^
Fold 1 (main)	22	660	14.28	16.92	0.86
Fold 2	21	630	15.63	19.47	0.86
Fold 3	21	630	13.74	16.31	0.86
Fold 4	21	630	16.21	20.05	0.86
Fold 5	22	660	14.89	18.86	0.85
Mean ± SD	-	-	14.95 ± 1.00	18.32 ± 1.63	0.858 ± 0.004

Note: Metrics were calculated at the animal level by averaging all image-pair predictions from the same animal. Values in the last row are reported as mean ± standard deviation across five folds.

### Appendix A.5. Ablation Results on the Validation Set Under Different Random Seeds

**Table A4:** Detailed validation results of different model variants under three random seeds.

Model	Seed	MAE/kg		RMSE/kg	
		Image-Level	Animal-Level	Image-Level	Animal-Level
ResNet50	42	13.19	11.37	20.20	16.92
	2024	14.32	12.06	21.13	17.84
	3407	13.27	11.41	20.16	17.01
MCA + ResNet50	42	14.32	11.82	18.89	16.54
	2024	13.46	11.58	19.73	17.36
	3407	13.72	11.13	19.25	16.98
CA + ResNet50	42	14.68	12.02	19.47	17.02
	2024	14.43	12.77	20.31	17.89
	3407	14.96	12.29	19.88	17.43
CBAM + ResNet50	42	12.84	11.16	18.17	17.55
	2024	13.59	11.68	19.22	16.71
	3407	13.17	11.25	18.86	16.24
CBAM + ResNet50 + MCA	42	12.76	9.74	18.13	14.91
	2024	13.48	10.42	18.39	15.21
	3407	12.05	10.03	18.39	15.21
CBAM + ResNet50 + SE	42	11.98	9.01	17.96	14.34
	2024	11.21	9.58	18.31	14.02
	3407	12.53	9.24	17.88	14.67
